# Drinking and Drinking-Related Problems Among Alaska Natives

**Published:** 1998

**Authors:** Bernard Segal

**Affiliations:** Bernard Segal, Ph.D., is director of the Center for Alcohol and Addiction Studies, University of Alaska, Anchorage, Alaska

In 1996, the most recent year for which data are available, alcohol consumption in Alaska was 2.63 gallons per person^1^; only eight States had higher rates, many of which are attributable to cross-border alcohol sales (Williams et al. 1998). Alaska’s consumption rate has been among the highest in the Nation in each year for which statistics exist. Although heavy alcohol use in Alaska is not restricted to Alaska Natives, alcohol abuse and its consequences are disproportionately high among this group, which constitutes approximately 15.7 percent of Alaska’s total population (Alaska Department of Labor 1996).

One theory to explain the high rates of alcohol use among this special population faults the rapid industrialization that has taken place in Alaska. For many Alaska Natives, conflicts involving cultural identity as well as behavioral and lifestyle problems have resulted from adjusting to the rapid cultural changes. One way of coping with those feelings, particularly for younger Alaska Native men and women, may be to drink alcohol (Segal 1999).

This sidebar reviews what is known about alcohol use and alcohol-related problems among Alaska Natives. Directions for future research on preventing and treating alcohol abuse among this population also are discussed.

## Alcohol-Related Violence and Death Among Alaska Natives

Since the late 1980s, Alaska has been among the five States with the highest annual rates of child abuse, accidental death, assaults, rape, and suicide, all of which have been linked to alcohol abuse (Brems 1996). For example, 25 percent of all deaths in Alaska are alcohol-related (Alaska Department of Health and Social Services [ADHSS] 1994). More recently, of the 192 Native deaths (from any cause) that occurred in rural Alaska between 1990 and 1993, 128 (66.6 percent) were found to be alcohol-related (i.e., the deceased had a blood alcohol concentration [BAC] of 0.08 or higher) (Demer 1997). In addition, Alaska Native men and women exceed other ethnic groups in Alaska with respect to alcohol-related problem behaviors, such as arrests for driving while intoxicated (DWI), alcohol-related accidents and injuries from automobile crashes, fishing-related accidents, and other causes of injury (ADHSS 1994).

Although all Alaskans have a higher risk of dying by accident or suicide compared with those in the lower 48 States, the rates are notably high for Alaska Natives. Suicides in Alaska have exceeded national rates for more than 20 years (Berman and Leask 1994). Hlady and Middaugh (1988) reported that the percentage of suicides that were alcohol-related in Alaska was almost twice the national average during the period 1983–1984 and that the percentage was significantly higher among Alaska Natives than among non-Natives (Hlady and Middaugh 1988).

## Alcohol-Related Health Problems Among Alaska Natives

Alaska Natives have unusually high rates of drinking, which results in many health problems. Hisnanick (1992) reported that between 1980 and 1987, Alaska ranked fifth among 11 Indian Health Service sites for alcohol-related illnesses and symptoms, such as liver cirrhosis, delirium tremens (DTs), and pancreatitis.

Another alcohol-related health problem is fetal alcohol syndrome (FAS), which appears to occur with higher frequency among Alaska Natives than among other populations. Weeks (1989) reported an FAS rate among Alaska Natives of 5.2 cases per 1,000 births, with regional variations of from 2.7 to 20.6 cases per 1,000 births. In comparison, the FAS rate for the United States ranges from 1 to 3 cases per 1,000 births (May 1996). Although the reliability of some of Alaska’s FAS data has recently been questioned (Segal 1999), the problem remains serious: In 1994, 39 percent of pregnant Alaska Native women were estimated to be at risk for delivering a baby prenatally exposed to alcohol or other drugs (Alaska Area Native Health Service 1995).

## Correlates of Drinking Among Alaska Natives

The high rates of violence and health problems attest to the seriousness of drinking and its effects among Alaska Natives (Brems 1996; Segal 1983*a*, 1983*b*, 1990, 1991*a*, 1991*b*, 1999; Segal and Hesselbrock 1997). Relatively little information, however, has been reported about the factors that might underlie those problems. The next sections describe specific areas in which research is needed to better understand alcohol use and abuse among Alaska Natives; those areas include genetics, quantity of consumption, behavioral and other correlates, and the possible role that a loss of Native culture may play in problem drinking.

### Genetics

Until recently, it was believed that Alaska Natives were relatively recent descendants of East Asian ancestors. If that theory were accurate, one would expect Alaska Natives to possess a particular variant of a specific gene that has been linked to the alcohol-induced flushing reaction^2^ observed in some Asians after drinking (Shibuya and Yoshida 1989; Singh et al. 1989). This unpleasant reaction is believed to help mitigate against heavy drinking and alcoholism (Thomasson et al. 1991). A series of studies (Avksentyuk et al. 1994, 1995; Segal et al. 1998; Thomasson et al. 1992), however, found that Alaska Natives do not resemble Asians with respect to this genetic trait. These findings and other research (Segal et al. 1998; Chen et al. 1997) suggest that the genetic characteristics that may “protect” some people against alcoholism are not present in Alaska Natives. It is unknown, however, if Alaska Natives have a genetic factor that may place them at particular risk for developing alcoholism. Given the disproportionately high numbers of alcohol problems in this population it is important that this group be included in studies searching for a genetic link to alcoholism.

### Quantity of Alcohol Consumed

Alaska Natives who drink heavily may consume greater quantities of alcohol per drinking session than their non-Native counterparts. Segal (1991*a*) studied repeat users of an Anchorage “sleep-off center” (i.e., a shelter where homeless inebriates could sleep off their intoxication) and found that the average BAC of the Alaska Natives who entered the facility during the study period was significantly higher than that of the Caucasians entering the shelter (0.186 versus 0.137).^3^ No differences in BACs were found between genders for either ethnic group. Additional research is necessary to verify that the pattern of alcohol consumption among Alaska Natives is different from that found in other populations.

### Behavioral and Other Factors

Survey data, which compare common manifestations of alcohol abuse among Alaska Natives and other ethnic groups, have been compiled. The table on page 278 compares Segal and Hesselbrock’s (1997) study group of Alaska Natives with other ethnic groups on selected behavioral and psychiatric characteristics; it shows that in many categories, Alaska Natives present a more severe set of physical and social complications. No research has focused on whether certain drinking behaviors are specific to Alaska Natives. Moreover, the methodology of the alcohol studies that have been completed in Alaska prohibits direct comparisons among ethnic groups (Hesselbrock et al. 1997).

### Family Violence

Native women in Alaska face a much higher risk of violence than do women nationwide (Berman and Leask 1994). The severity and nature of the violence is consistent with other research showing a relationship between being victimized and drinking (e.g., Miller et al. 1993). Studies are needed to determine the effect that such family violence has on children (e.g., does it place children at risk for abuse and neglect as well as increase the chance that they too will abuse alcohol later in life?).

### Cultural Factors

The cultures of indigenous Alaskans have been radically modified by the influx of Russians, Anglo-Europeans, and other people, who have imposed new customs, traditions, and economic systems. Over the past 25 to 30 years, the development of the oil industry has spurred Alaska Natives’ transition from a subsistence to a cash economy. The resulting alterations in family roles, community functions, and other aspects of culture may play a role in Alaska Natives’ use of alcohol. Research will help to determine the relationship between changing cultural mores and increased alcohol use.

**Table:** Comparison of Alaska Natives With Other Ethnic Groups on Selected Characteristics

	Ethnic Group (%)							
	Alaska Native		Caucasian		African-American		Hispanic	
	Male (*n*=141)	Female (*n*=120)	Male (*n*=1,087)	Female (*n*=443)	Male (*n*=285)	Female (*n*=119)	Male (*n*=74)	Female (*n*=25)
	
**Alcohol-Related Violence**								
Arguments	94.3	95.8	84.7	80.7	84.6	80.7	86.5	96.0
Threw/hit things	88.7	80.8	69.1	65.5	59.3	52.1	64.9	76.0
Hit family	58.9	65.0	33.8	37.0	37.2	37.0	35.1	48.0
Hit others	46.1	49.2	25.8	28.2	34.4	28.6	29.7	46.1
Physical fights	88.7	75.0	66.6	46.3	68.4	42.9	71.7	72.0
**Serious Alcohol Symptoms**								
Morning drinking*	64.0	63.0	51.5	41.8	57.5	56.3	50.0	32.0
Delirium tremens	36.9	26.7	21.6	24.4	18.9	20.2	23.0	28.0
Seizures	7.8	6.7	6.7	4.1	4.9	9.2	12.2	4.0
Stomach problems	29.8	18.3	13.7	11.3	16.5	12.6	8.1	20.0
Liver disease	4.3	5.8	12.9	6.8	9.8	10.1	13.5	12.0
Pancreatitis	2.1	2.5	3.6	2.3	7.0	9.2	4.1	4.0
**Alcohol-Related Behavior Problems**								
Driving while intoxicated (DWI)	68.8	45.8	62.6	28.9	34.4	7.6	56.8	16.0
Arrests	73.8	69.2	46.6	23.9	44.6	29.4	45.9	28.0
Accident/injury	68.8	72.5	62.7	58.9	50.2	47.1	50.0	60.0
Reckless behavior	95.7	89.2	97.3	90.7	87.7	58.0	95.9	88.0
Drug Abuse/Antisocial Personality Disorder (ASPD)**								
Marijuana	62.5		42.8		39.6		45.5	
Cocaine	44.1		37.6		63.5		44.9	
Stimulants	14.6		23.4		10.7		29.3	
ASPD	33.3		20.1		20.3		34.3	

* The number of Alaska Native cases for this variable only is 50 males and 46 females.

** Percentages are for men and women combined.

The number of cases is the same as shown at the top of the table.

NOTE: The comparison groups were derived from consecutive admissions to alcohol residential treatment facilities who met both DSM–IV criteria for alcohol dependence and Feighner criteria for definite alcoholism The Feighner criteria (Feighner et al. 1972) were the first set of diagnostic criteria for alcoholism to be based on research rather than on subjective judgment and clinical experience. They were developed in the 1970s in response to perceived deficiencies in the first and second editions of the American Psychiatric Association’s *Diagnostic and Statistical Manual of Mental Disorders*.

SOURCES: Hesselbrock et al. 1998; Segal 1998.

## Treatment Issues

Clearly, the problems associated with abusive drinking and other drug use among Alaska Natives are severe. Sociocultural factors likely play an important role in drinking behavior. Alaska Natives may benefit from treatment that incorporates Native values and attitudes, although research is needed to determine whether culture-specific treatment programs are more effective than other programs. Preliminary research on a culturally oriented treatment program for Alaska Native women is encouraging (Segal 1998).

## Future Research Directions

Treatment and prevention of alcohol problems among Alaska Natives would be enhanced by research efforts in the following areas:

- The increases in alcohol abuse and other alcohol-related problems in Alaska correspond to a period of rapid growth and industrialization and a concomitant loss of Native cultural traditions. Research is needed to determine whether a cause-and-effect relationship exists between cultural loss and Alaska Natives’ alcoholism rates.
- Further research is needed to demonstrate how genetic factors may predispose Alaska Natives to alcohol problems.
- Research is needed to address the risk factors, behavioral correlates, and signs and symptoms of alcohol dependence that are specific to Alaska Natives.
- Studies are needed to refine our understanding of the severe behavioral manifestations of alcohol abuse among Alaska Natives and the connection of drinking with high rates of violence among this population. For example, intergenerational transmission of violent behavior is a serious problem in Alaska Native families. Future research should explore how to treat and prevent alcohol abuse among victims of physical and sexual abuse as well as investigate ways of breaking the cycle of violence.
- Studies must be undertaken to determine if incorporating cultural factors into treatment makes those approaches more effective. For instance, how does the increased training and employment of Alaska Native alcoholism counselors, as well as additional cultural training for non-Native counselors, affect treatment results with Alaska Native clients?
- Prevention-based approaches are important and can be enhanced by research to improve understanding of how cultural factors influence the initiation of drinking and drug taking or reinforce drinking behavior once it begins.

## Summary

Alcohol use has adversely affected many aspects of the Alaska Native community. To a large extent, overcoming the problem of alcohol abuse may require that Alaska Natives craft individual and community solutions to detrimental health, social, and economic conditions and instill new patterns of living that inhibit alcohol abuse. An example of this approach is the Alaska Federation of Natives’ “sobriety movement,” a grassroots campaign to promote sobriety that emphasizes traditional values and lifestyles. The use of “healing” or other traditional methods may help Alaska Natives both recover from the trauma of decades of cultural conflict and address alcohol problems in their communities.

## Footnotes

1 Per person alcohol consumption equals the total quantity of alcohol sold divided by the total population age 14 and over in the State.

2 The alcohol-induced flushing reaction consists of increased blood flow to the face, neck, and chest and is sometimes accompanied by increased heart rate, decreased blood pressure, and nausea.

3 The difference was statistically significant (*F* = 35.225, *df* = 1, *p* < 0.001).
